# Biodistribution of adeno-associated virus serotype 9 (AAV9) vector after intrathecal and intravenous delivery in mouse

**DOI:** 10.3389/fnana.2014.00042

**Published:** 2014-06-10

**Authors:** Daniel J. Schuster, Jaclyn A. Dykstra, Maureen S. Riedl, Kelley F. Kitto, Lalitha R. Belur, R. Scott McIvor, Robert P. Elde, Carolyn A. Fairbanks, Lucy Vulchanova

**Affiliations:** ^1^Departments of Neuroscience, University of MinnesotaMinneapolis, MN, USA; ^2^Department of Veterinary and Biomedical Sciences, University of MinnesotaSaint Paul, MN, USA; ^3^Departments of Genetics Cell Biology and Development, University of MinnesotaMinneapolis, MN, USA; ^4^Departments of Pharmaceutics, University of MinnesotaMinneapolis, MN, USA

**Keywords:** adeno-associated, AAV9, intrathecal, intravenous, CNS, DRG, trigeminal

## Abstract

Adeno-associated virus serotype 9 (AAV9)-mediated gene transfer has been reported in central nervous system (CNS) and peripheral tissues. The current study compared the pattern of expression of Green Fluorescent Protein (GFP) across the mouse CNS and selected peripheral tissues after intrathecal (i.t.) or intravenous (i.v.) delivery of equivalent doses of single-stranded AAV9 vector. After i.t. delivery, GFP immunoreactivity (-ir) was observed in spinal neurons, primary afferent fibers and corresponding primary sensory neurons at all spinal levels. Robust transduction was seen in small and large dorsal root ganglion (DRG) neurons as well as trigeminal and vagal primary afferent neurons. Transduction efficiency in sensory ganglia was substantially lower in i.v. treated mice. In brain, i.v. delivery yielded GFP-immunoreactivity (-ir) primarily in spinal trigeminal tract, pituitary, and scattered isolated neurons and astrocytes. In contrast, after i.t. delivery, GFP-ir was widespread throughout CNS, with greater intensity and more abundant neuropil-like staining at 6 weeks compared to 3 weeks. Brain regions with prominent GFP-ir included cranial nerve nuclei, ventral pons, cerebellar cortex, hippocampus, pituitary, choroid plexus, and selected nuclei of midbrain, thalamus and hypothalamus. In cortex, GFP-ir was associated with blood vessels, and was seen in both neurons and astrocytes. In the periphery, GFP-ir in colon and ileum was present in the enteric nervous system in both i.v. and i.t. treated mice. Liver and adrenal cortex, but not adrenal medulla, also showed abundant GFP-ir after both routes of delivery. In summary, i.t. delivery yielded higher transduction efficiency in sensory neurons and the CNS. The observation of comparable gene transfer to peripheral tissues using the two routes indicates that a component of i.t. delivered vector is redistributed from the subarachnoid space to the systemic circulation.

## Introduction

Adeno-associated virus serotype 9 (AAV9) vector has engendered considerable interest in its therapeutic development for treating neurological disorders (Dayton et al., 2012), in part because it has been heralded as capable of traversing the blood brain barrier to target the central nervous system (CNS), where it has been shown to transduce astrocytes and neurons (Foust et al., 2009; Gray et al., 2011; Samaranch et al., 2013). Enthusiasm for using AAV9-mediated gene therapy for global CNS transduction or targeting of specific neuronal populations has lead to investigations on the optimal route of administration to the CNS, scalability from rodent models to larger animal species, and appropriate promoters for broad distribution, cell-specific targeting, or improved duration of expression (Gray et al., 2011; Snyder et al., 2011; Federici et al., 2012; Samaranch et al., 2012, 2013; Dirren et al., 2014). Whereas the intravenous route of delivery of AAV9 has been examined extensively and successfully utilized in models of neurological disorders (Foust et al., 2009; Fu et al., 2011; Ruzo et al., 2012; Garg et al., 2013; Shen et al., 2013; Yamashita et al., 2013; Elmallah et al., 2014), AAV9 administration into the CSF has been employed less frequently in rodents (Snyder et al., 2011; Haurigot et al., 2013; Hirai et al., 2014), and the biodistribution of the vector following intrathecal delivery has not been fully characterized. This direct CNS route of administration offers advantages such as reduction in the vector dose required for CNS transduction, and potentially restricted distribution, minimizing off-target effects (Gray et al., 2011, 2013; Snyder et al., 2011; Samaranch et al., 2013).

We and others have shown that delivery of AAV vectors within the lumbar intrathecal space leads to expression of green fluorescent protein (GFP) in dorsal root ganglion (DRG) neurons as well as brain and peripheral tissues (Storek et al., 2008; Towne et al., 2009; Mason et al., 2010; Vulchanova et al., 2010; Schuster et al., 2013). Although AAV9-mediated transduction of primary sensory neurons has been previously reported in rodents and non-human primates (Hirai et al., 2012, 2014; Gray et al., 2013), the pattern of AAV9-driven transgene expression in sensory ganglia has not been thoroughly examined. In the present study, we evaluated the ability of AAV9 to transduce primary sensory neurons as well as its distribution to brain regions due to rostral flow of CSF when delivered intrathecally by direct lumbar puncture. In addition, we examined the extent of GFP expression in selected peripheral organs and tissues to assess potential systemic redistribution of the vector or transport of the transgene in peripheral nerves. We hypothesized that, in comparison to titer-matched intravenous delivery, intrathecal administration of AAV9-GFP will result in enhanced transduction of primary sensory neurons, spinal cord and brain and reduced transduction in peripheral tissues and organs.

## Materials and methods

### Vector

AAV9-GFP was purchased from the vector core at the University of Pennsylvania (AAV9.CB7.Cl.eGFP.WPRE.rBG; cat # AV-9-PV1963).

### Animals

Experimental subjects were 20–25 g adult male C57BL/6 mice (6–8 weeks old at the time of treatment; Harlan, Madison, WI). All experiments were reviewed and approved by the Institutional Animal Care and Use Committee (IACUC) of the University of Minnesota.

### Injections

For the comparison of intrathecal (i.t.) and intravenous (i.v.) delivery in the presence or absence of mannitol, 16 mice were divided into four groups: mannitol +i.v. injection, mannitol + i.t. injection, i.t. injection only, and i.v. injection only. *Mannitol pretreatment*: Mannitol is a hyperosmotic agent that enhances intraparenchymal diffusion following intracerebroventricular (i.c.v.) injection of AAV particles (Mastakov et al., 2002; Fu et al., 2003). Previously, we observed that mannitol pretreatment enhanced AAV5-mediated transduction of primary sensory neurons (Vulchanova et al., 2010) and in the present study sought to determine whether it also impacts AAV9 particle distribution. Subjects were injected via the tail vein with 25% mannitol solution (200 μL) 20 min prior to i.t. or i.v. injection of AAV9-GFP. *Intravenous delivery:* AAV9-GFP (~3.3 × 10^11^ vector genomes) was diluted 10-fold in sterile saline for a final injectate volume of 100 μL, injected via the tail vein. *Intrathecal delivery:* AAV9-GFP (10 μL containing ~3.3 × 10^11^ vector genomes) was delivered by direct lumbar puncture in awake mice as previously described (Hylden and Wilcox, 1981; Fairbanks, 2003; Vulchanova et al., 2010). In addition, 4 mice were injected i.t. with a reduced AAV9-GFP dose (~6.6 × 10^9^ vector genomes) following mannitol pretreatment. Three weeks after injection, mice were subjected to transcardial perfusion fixation. Prior to the 3-week endpoint one mouse from the mannitol + i.t. injection group was euthanized due to seizures. Finally, four mice received mannitol + i.t. injection (1.73 × 10^11^ vector genomes) and were evaluated 6 weeks after vector delivery.

### Immunohistochemistry

All animals were sacrificed by perfusion fixation as previously described (Vulchanova et al., 1997). Briefly, animals were deeply anaesthetized and perfused with a solution of calcium-free tyrodes solution (in mM:NaCl 116, KCl 5.4, MgCl_2_·6H_2_0 1.6, MgSO_4_·7H_2_O 0.4, NaH_2_PO_4_ 1.4, glucose 5.6, and NaHCO_3_ 26) followed by fixative (4% paraformaldehyde and 0.2% picric acid in 0.1 M phosphate buffer, pH 6.9). Tissues were removed and stored in PBS containing 10% sucrose and 0.05% sodium azide at 4°C until further use. Sections were cut at 14 μm thickness and thaw mounted onto gel-coated slides. Tissue sections were incubated for 1 h at room temperature in diluent (PBS containing 0.3% Triton, 1% BSA, 1% normal donkey serum) and then incubated overnight at 4°C in primary antisera diluted in the same solution. Primary antibodies used were: chicken anti-GFP, 1:1000 (Abcam, cat# 13970; specificity confirmed by lack of labeling in the absence of AAV9-GFP treatment); rabbit anti-GFP, 1:500 (Invitrogen; Eugene, OR; specificity confirmed by lack of labeling in the absence of AAV9-GFP treatment); rabbit anti-GFAP, 1:1000 (ICN, Costa Mesa, CA; pattern of labeling equivalent to that obtained with a mouse anti-GFAP antibody and consistent with exclusive staining of astrocytes); rabbit anti-Iba1 [Wako Chemicals USA, Inc.; cat# 019-1974; specificity established based on Western blot (Imai et al., 1996) and colocalization with other microglial markers (Ito et al., 1998)]; rabbit anti-calcitonin gene related peptide (CGRP), 1:1000 (ImmunoStar, Hudson WI, cat# 24112; specificity established by preabsorption with CGRP and unrelated peptides—manufacturer's information); guinea pig anti-P2X3, 1:1000 [the generation and specificity of this antiserum is described in (Vulchanova et al., 1997)]; rat anti-substance P (SP), 1:100, [Abd Serotec, Oxford, UK; the specificity of this antibody is described in (Riedl et al., 2009)]; rat anti-CD31, 1:300 (BD Pharmingen, cat# 557355; pattern of labeling consistent with staining of blood vessels). After rinsing with PBS, sections were incubated for 1 h at room temperature with appropriate combinations of Cy2-, Cy3-, and Cy5- (1:300) conjugated secondary antisera (Jackson ImmunoResearch, West Grove, CA). Sections were rinsed again, and in some cases, were also incubated with DAPI nucleic acid stain for 3–5 min, 300 nM (Invitrogen; Eugene, OR) or NeuroTrace (Invitrogen) according to manufacturer's instructions. Following the final rinses, sections were cover-slipped using glycerol and PBS containing 0.1% p-phenylenediamine (Sigma). Tissues from a naïve mouse processed in parallel with the AAV9-GFP treated tissues were included in all immunohistochemical experiments to control for non-specific staining of the GFP antibody.

### Microscopy

Anatomical analysis was based on the “The Mouse Brain In Stereotaxic Coordinates,” Second Edition (Paxinos and Franklin, 2001) and the Mouse Brain Library C57BL/6 mouse brain atlas (Rosen et al., 2000). Images were collected on an Olympus Fluoview 1000 confocal microscope with associated software. For comparisons of tissues from i.t. and i.v. treated mice, images were collected using the same microscope setting with a few exception where adjustments were necessary to allow detection of labeling in i.v. treated tissues or avoid saturation in i.t. treated tissues. Similarly image adjustments for contrast, brightness and color in Adobe Photoshop were performed in parallel for i.t. and i.v. treated tissues.

### Quantification of GFP expression in sensory ganglia

For each ganglion, 7–8 non-overlapping images taken across 4–5 tissue sections, which were spaced by at least 56 μm, were used for analysis. Neurons were outlined based on NeuroTrace Nissl-like labeling, and only cells with a visible nucleus were counted. Measurements of GFP-ir fluorescence intensity and cell area were obtained using Image J software. The intensity measurements of unlabeled cells were used to determine the labeling threshold for unbiased identification of GFP-positive neurons. For each ganglion the number of GFP-positive neurons was determined as percentage of all neurons in the sampled sections. The data are expressed as Mean ± Standard Error. Statistical analysis was performed using GraphPad Prism 5 software. Comparisons were made using One-Way ANOVA followed by Bonferroni *post-hoc* test or by *t*-test.

### Analysis of histopathology

Routine hematoxylin and eosin staining was performed on cryosections mounted on gel-coated slides. For each animal in the 3-week treatment groups, 4–6 section per ganglion were evaluated by a trained, blinded observer and scored based on lesion number and severity. Lesion scoring: 0, No gross lesions; 1, Increased mononuclear cells without neuron loss; 2, Distinct nodular aggregate of mononuclear cells replacing one neuron; 3, Discrete nodular aggregates of mononuclear cells replacing more than one neuron; 4, Mononuclear infiltrates and nodular aggregates replacing at least three neurons. For each ganglion, average lesion score was calculated based on the lesion score of each of the examined sections. The data is reported as mean ± SE of the average lesion score for each experimental group. Tissues included dorsal root ganglia, spinal cord, liver, skeletal muscle, ileum, and colon.

## Results

### AAV9-mediated transduction of primary sensory neurons and their CNS projection targets

**Figure 1:**
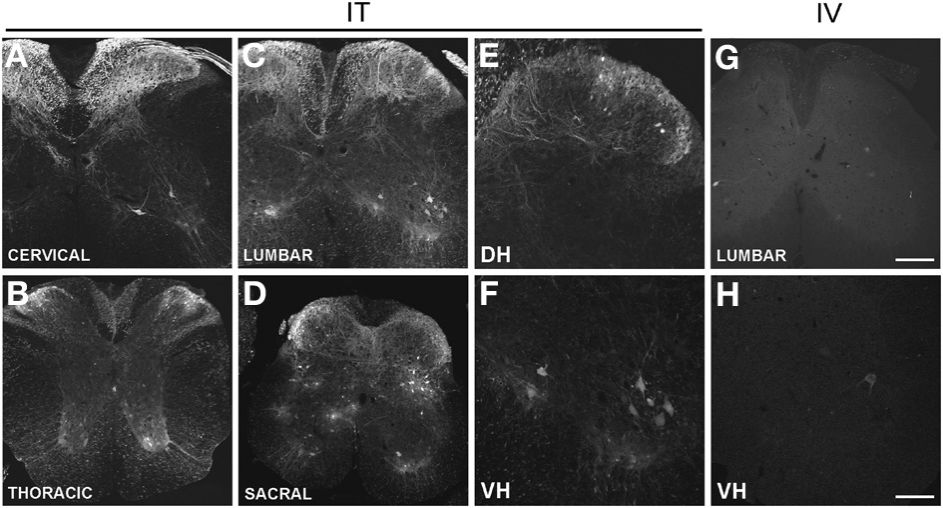
**(A–H)** Localization of GFP-ir in the spinal cord 3 weeks after i.t. (IT) or i.v. (IV) delivery of AAV9-GFP without mannitol. **(A–D)**, GFP-ir in cervical **(A)**, thoracic **(B)**, lumbar **(C)**, and sacral **(D)** spinal cord after i.t. delivery. **(E,F)** GFP-ir in dorsal (DH, **E**) and ventral (VH, **F**) horn of lumbar spinal cord after i.t. delivery. **(G,H)** GFP-ir in lumbar spinal cord **(G)** and ventral horn (VH, **H**) after i.v. delivery. Scale bars: 200 μm in **(A–D)**, and **(G)**; 100 μm in (**E,F,H**).

**Figure 2:**
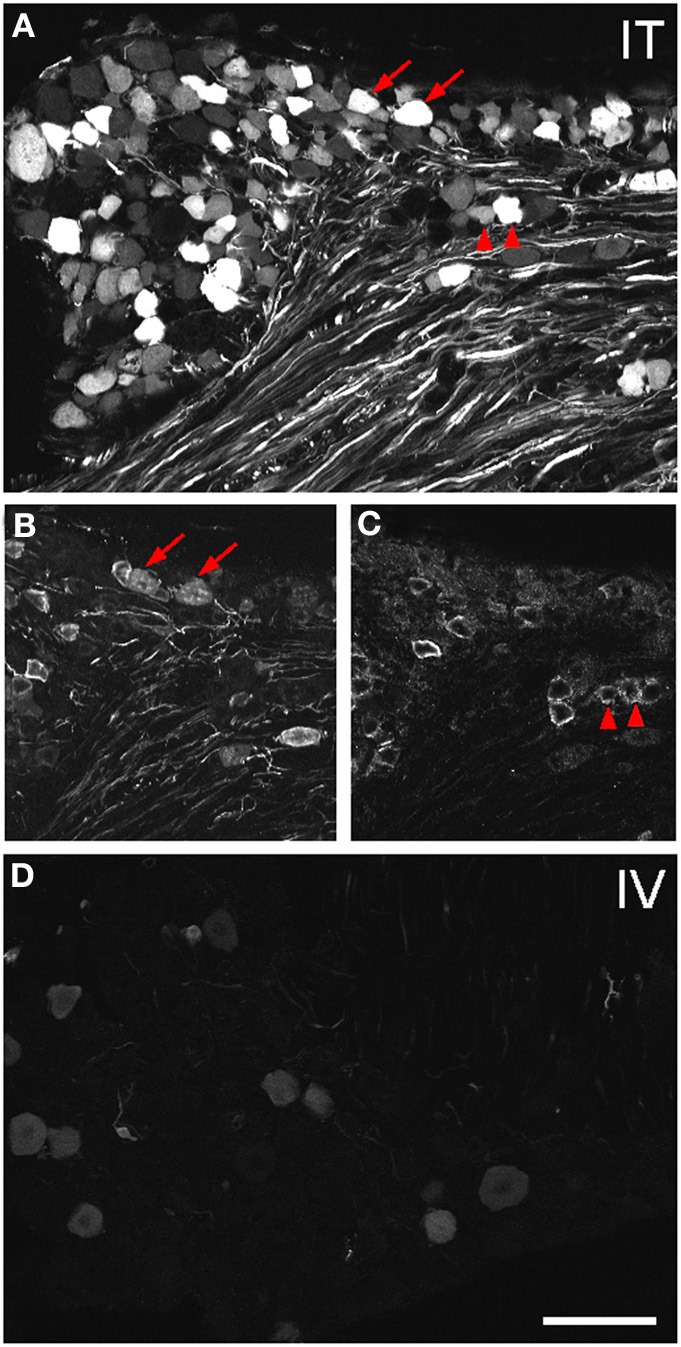
**Localization of GFP-ir in lumbar DRG 3 weeks after i.t. or i.v. delivery of AAV9-GFP without mannitol. (A–C)** GFP-ir in L4 DRG after i.t. delivery and its relationship to CGRP- **(B)** and P2X3-ir **(C)** Arrows and arrowheads indicate colocalization of GFP- and CGRP-ir and GFP- and P2X3-ir, respectively. **(D)** GFP-ir in L4 DRG after i.v. delivery. Scale bar: 100 μm.

Three weeks after i.t. AAV9-GFP delivery, intense GFP-ir was observed at all levels of spinal cord (Figures 1A–D). GFP-ir fibers were abundant in dorsal horn and dorsal columns, and labeling was seen in neuronal cell bodies in both dorsal and ventral horn (Figures 1E,F). In contrast, i.v. delivery of the viral vector resulted in limited labeling of fibers in dorsal horn and isolated cell bodies in ventral horn (Figures 1G,H). Consistent with the observations in spinal cord, GFP-positive neurons were present in DRG (Figure 2). Quantitative image analysis indicated that 73 ± 12% of L4 DRG neurons were GFP-positive after i.t. delivery (range 47–95%, *n* = 4), compared to 9 ± 1% after i.v. delivery (range 7–10%, *n* = 4) (Figure 4A). Mannitol pretreatment did not significantly enhance the transduction of DRG neurons by AAV9-GFP after i.t. or i.v. delivery (Figure 4A). Reduction in the number of viral particles delivered i.t. (50× dilution) correspondingly lead to reduction of the number of labeled neurons to 8 ± 4% (range 2–15%, *n* = 4). Analysis of the size distribution of GFP-positive DRG neurons suggested preferential transduction of large-diameter neurons, particularly after i.t. delivery of the 1/50 dose and after i.v. delivery (Figure 4B). However, i.t. delivery of full-strength AAV9-GFP resulted in substantial transduction of small-diameter DRG neurons (64 ± 17% of all small-diameter neurons, range 27–94%; Figure 4B), which was also illustrated by the frequent colocalization of GFP-ir with the markers of peptidergic and non-peptidergic small-diameter neurons, CGRP and P2X3 (Figures 2A–C).

**Figure 3:**
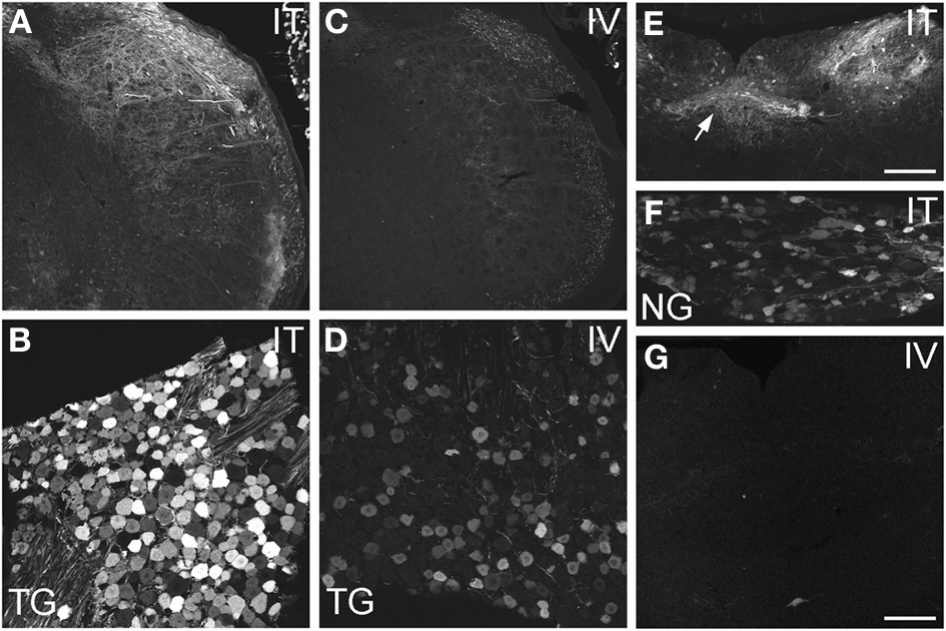
**Localization of GFP-ir in spinal trigeminal nucleus and nucleus of the solitary tract (NTS) and their corresponding sensory ganglia. (A–D)** GFP-ir in the spinal trigeminal nucleus **(A,C)** and trigeminal ganglion neurons **(B,D)** after i.t. **(A,B)** or i.v. **(C,D)** delivery. **(E–G)** Localization of GFP-ir in NTS (arrows, **E**) and the nodose ganglion **(F)** after i.t. delivery, and in NTS after i.v. delivery **(G)**. Detection of labeling in G required higher magnification and laser power than in E. Scale bars: 200 μm in **(A,C,E)**; 100 μmin **(B,D,F,G)**.

**Figure 4:**
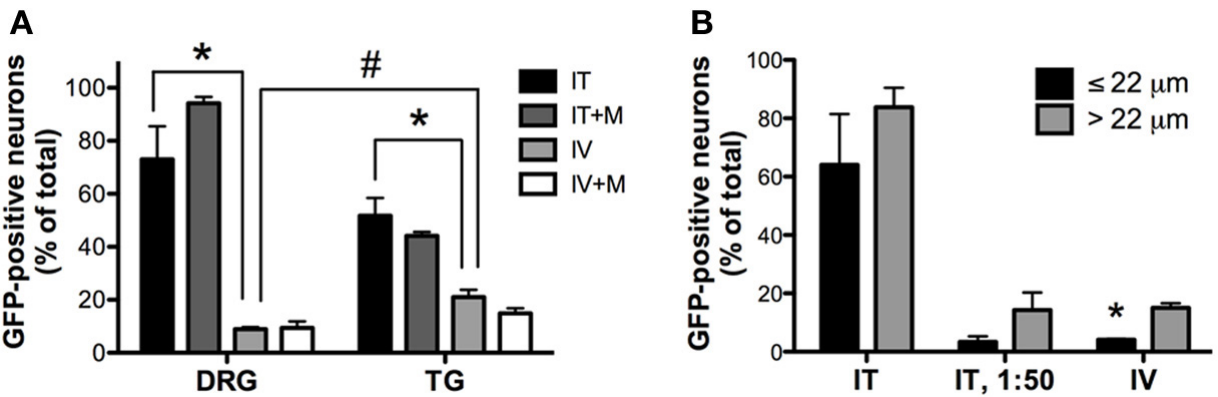
**Quantitative image analysis of the transduction of sensory neurons. (A)** Analysis of the proportions of GFP-positive DRG and trigeminal ganglion (TG) neurons [*n* = 4 for i.t. (IT) and i.v. (IV) groups; *n* = 2 for i.t. and i.v. groups with mannitol pretreatment (IT+M and IV+M, respectively)]. ^*^, indicates significant difference between i.t. and i.v. treatment groups in DRG and TG (*p* < 0.005; One-Way ANOVA for each type of ganglia followed by Bonferroni *post-hoc* test). #, indicates significant difference between DRG and TG in the i.v. treatment group (*p* < 0.01; *t*-test). **(B)** Analysis of the proportions of small-diameter (≤22 um) and large-diameter (>22 um) GFP-positive DRG neurons. ^*^, indicates significant difference between small- and large-diameter DRG neurons in the i.v. treatment group (*p* < 0.005; paired *t*-test).

AAV9-GFP transduction was also observed in the trigeminal sensory system (Figures 3A–D). Similar to spinal cord, GFP labeling in the spinal trigeminal tract and nucleus was substantially more abundant after i.t. delivery compared to i.v. delivery (Figures 3A,C). In trigeminal ganglia, GFP-ir was present in 52 ± 7% (range 37–69%, *n* = 4) and 21 ± 3% (range 15–27%, *n* = 4) of neurons in the i.t. and i.v. treatment groups, respectively (Figure 4A). Notably, the number of GFP-positive neurons after i.v. delivery was significantly higher in trigeminal ganglia compared to DRG (Figure 4A). As in DRG, mannitol pretreatment did not enhance the transduction of trigeminal sensory neurons. Finally, in addition to DRG and trigeminal ganglia, in i.t. treated mice GFP-ir was also seen in the vagal sensory system (Figures 3E–G). Labeling in the solitary tract and its associated nucleus was seen throughout their rostral-caudal extent (caudal medulla shown in Figure 3E). GFP-ir was also present in neurons of the nodose ganglion in i.t. treated mice. In i.v. treated mice detection of faint GFP-ir in the region of the solitary tract and nucleus was only seen using higher magnification and laser power (Figure 3G).

**Figure 5:**
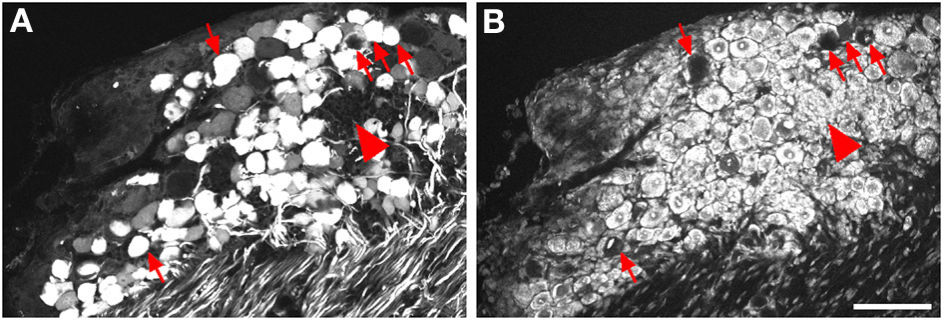
**Labeling for GFP (A) and NeuroTrace (B) shows GFP-positive neurons devoid of NeuroTrace staining (arrows) and clusters of satellite cell nuclei (arrowhead) indicative of gliosis**. Scale bar: 100 μm.

In the course of quantitative image analysis, we noted abnormal Nissl labeling, visualized by NeuroTrace, in some sections. This included intensely GFP-labeled neurons that were devoid of NeuroTrace staining (Figures 5A,B, arrows) as well as morphology consistent with satellite cell proliferation (Figures 5A,B, arrowhead). Subsequent histopathological evaluation revealed the presence of mononuclear aggregates and infiltrates in DRG, and to a lesser extent in trigeminal ganglia, of mice receiving i.t. AAV9. Lesions ranged in severity from infrequent, discrete nodules of residual satellite glial cells replacing solitary neurons to exuberant satellite glial cell proliferation with multifocal neuronal necrosis and loss. The mean lesion score for i.t. treated mice was 1 ± 0.8 for DRG, and 0.03 ± 0.2 for TG, and for i.t.+mannitol treated mice was 2.5 ± 1.2 for DRG and 0.8 ± 0 for TG (for the i.v. treatment groups the mean score was <0.05). A cursory examination of other tissues, including spinal cord, liver, skeletal muscle, and gut, did not reveal additional lesions.

### AAV9-mediated GFP expression in the CNS

**Figure 6:**
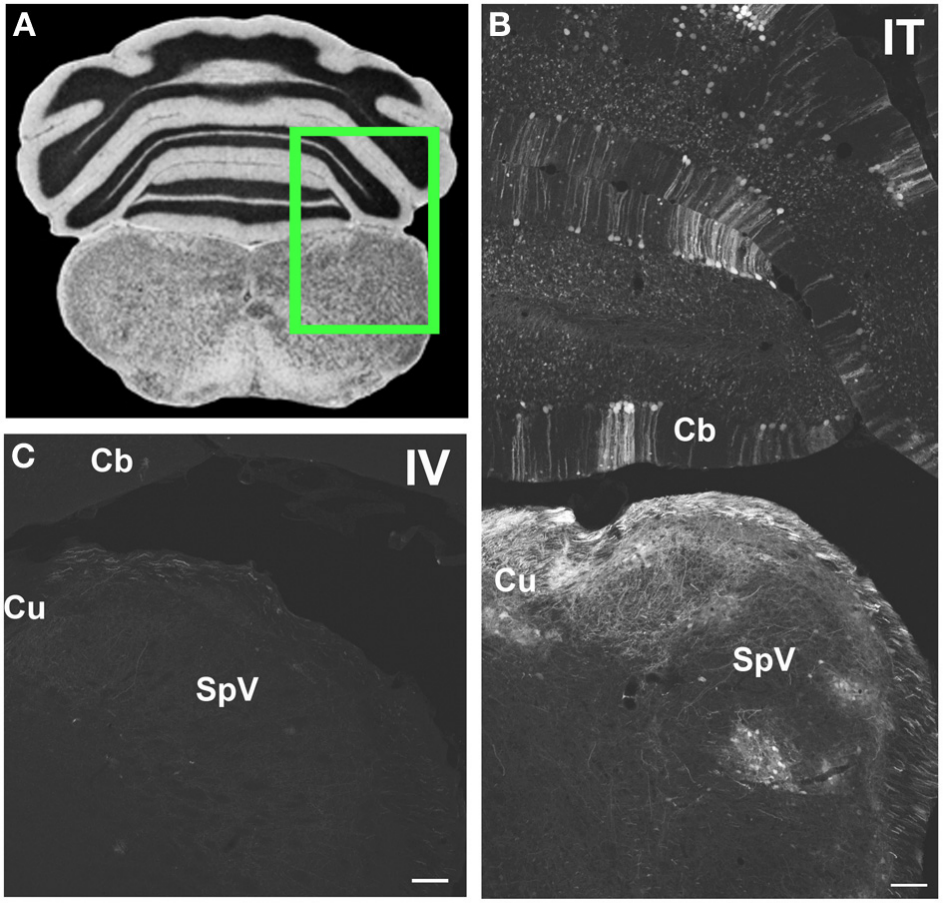
**Representative images of GFP-ir in medulla and cerebellum 3 weeks after i.t. (IT) or intravenous i.v. (IV) AAV9-GFP delivery. (A)** Coronal section labeled with cresyl violet was selected from the Mouse Brain Library (Rosen et al., 2000) C57BL/6 mouse brain atlas. Inset indicates approximate relative location of images shown in **(B,C)**. **(B)** GFP-ir associated with cuneate nucleus (Cu), spinal nucleus of V (SpV), and cerebellar cortex (Cb) after i.t. delivery. **(C)** Limited GFP-ir in cuneate and spinal trigeminal nucleus after i.v. delivery. Scale bar: 100 μm.

**Figure 7:**
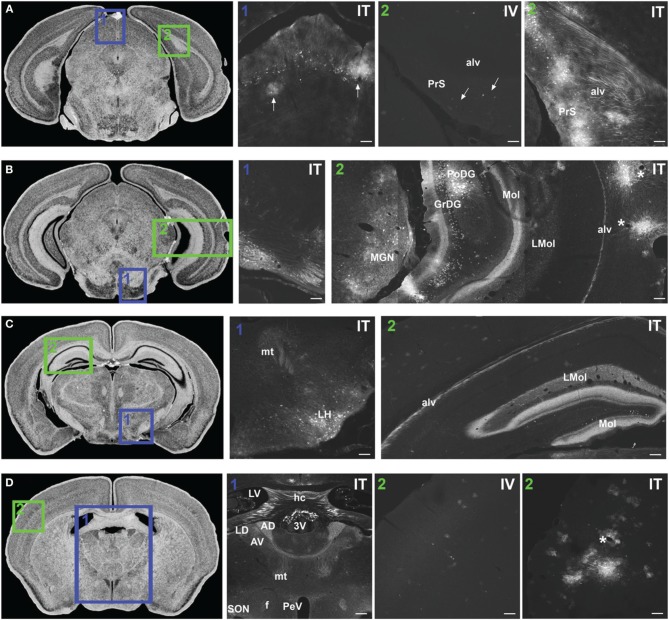
**Representative images of GFP-ir in midbrain and diencephalon**. Coronal sections labeled with cresyl violet were selected from the Mouse Brain Library (Rosen et al., 2000) C57BL/6 mouse brain atlas. Insets indicate approximate relative location of images shown in the associated panels. **(A) Panel A1:** GFP-ir in superior colliculus. Neuronal cell bodies appear as a band of puncta in the internal gray layer of the superior colliculus. Arrows indicate clusters of astrocytes associated with blood vessels. **Panel A2:** Intense GFP-ir is present in presubiculum (PrS) and alveus (alv) of the hippocampal formation after i.t. delivery, whereas after i.v. delivery labeling is limited to isolated cells (arrows). **(B) Panel B1:** GFP-ir in pontine nuclei and transverse pontine fibers. **Panel B2:** GFP-ir in the medial geniculate nucleus (MGN) of the thalamus, hippocampal formation, and temporal cortex. GFP-ir is present in many neurons of the polymorph (PoDG) and granular layers (GrDG) of the dentate gyrus as well as in nerve fibers of the molecular layer of dentate gyrus (Mol), stratum lacunosum-moleculare (LMol), and alveus (alv). Asterisks indicate blood vessels in proximity to clusters of labeled cells in cortex. **(C) Panel C1:** GFP-ir in lateral hypothalamus (LH) and mammilothalamic tract (mt). **Panel C2:** GFP-ir in the rostral-dorsal hippocampal formation. The molecular layer of the dentate gyrus (Mol), stratum lacunosum-moleculare (LMol), and alveus (alv) are highlighted by GFP-ir. **(D) Panel D1:** GFP-ir in the lateral dorsal nucleus (LD) of thalamus and in axons from the mammillo-thalamic tract (mt) entering portions of the anterior nuclear complex (AD and AV). Labeling is also present in the fornix (f), paraventricular (PeV), and supraoptic (SON) nuclei of hypothalamus, the hippocampal commissure (hc), and choroid plexus of the third (3V) and lateral ventricles (LV). **Panels D2 IV** and ITillustrate the difference in cortical expression between delivery routes. Asterisk indicates a blood vessel surrounded by GFP-ir cells. Scale bars: 100 μm in all panels except **Panel D1**, for which the scale bar is 250 μm.

**Figure 8:**
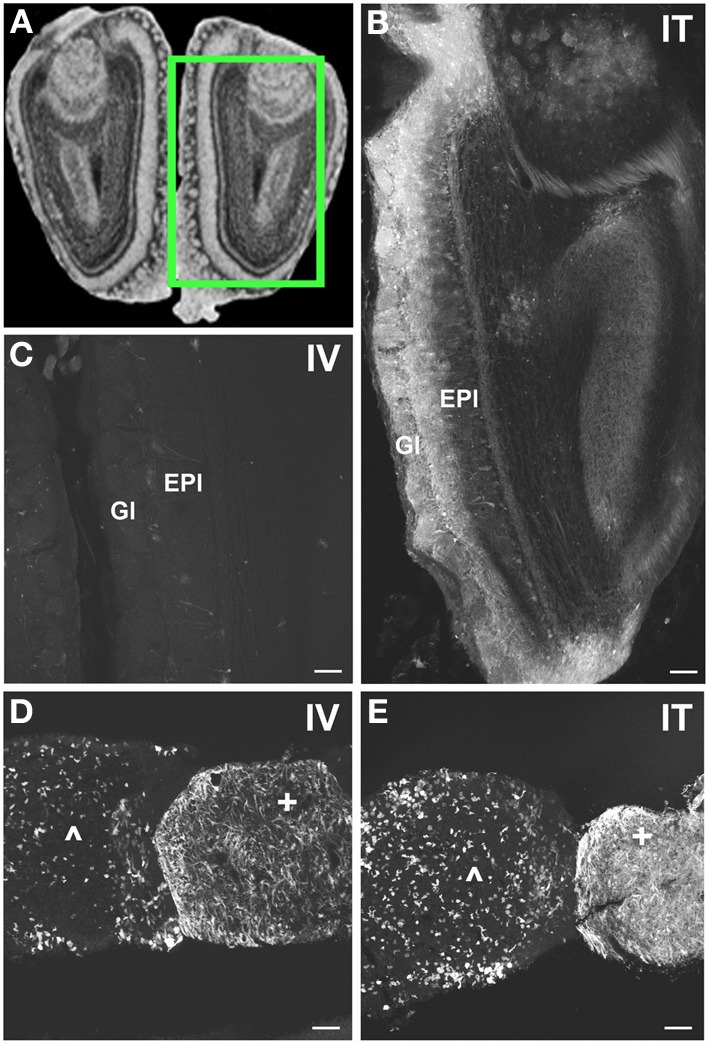
**Localization of GFP-ir in olfactory bulb and pituitary gland. (A)** Coronal section labeled with cresyl violet was selected from the Mouse Brain Library (Rosen et al., 2000) C57BL/6 mouse brain atlas. Inset indicates approximate relative location of images shown in **(B,C)**. **(B)** After i.t. delivery, GFP-ir was found throughout the olfactory bulb, but was most intense in the glomerular (Gl) and external plexiform (EPl) layers. **(C)** Limited GFP-ir in olfactory bulb after i.v. delivery. **(D,E)** Localization of GFP-ir in pituitary. Labeling in anterior pituitary (^∧^) was comparable between delivery routes; however, labeling in posterior pituitary (+) appeared more intense after i.t. **(D)** delivery compared to i.v. **(E)**. Scale bars: 100 μm.

**Figure 9:**
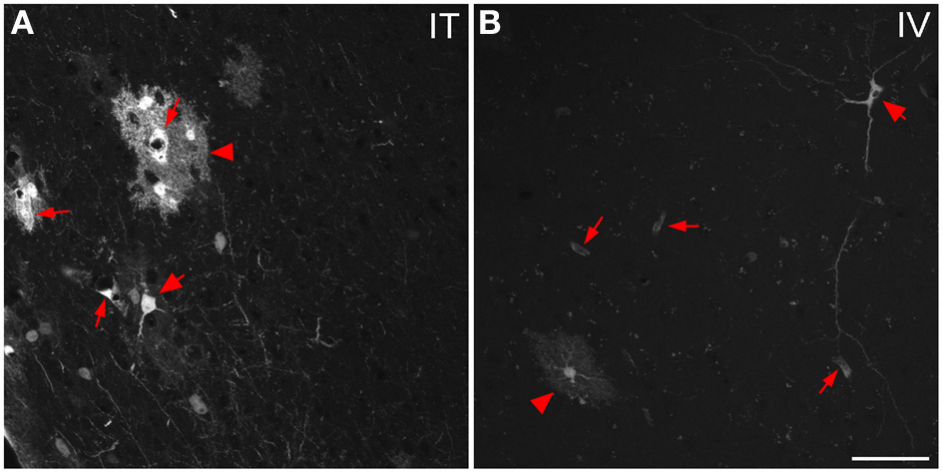
**Representative images of GFP-ir cells in cortex. (A)** After i.t. administration, intensely labeled clusters of cells were observed in close proximity to blood vessels, which are indicated by small arrows. The morphology of labeled cells was consistent with astrocytes (arrowheads) and neurons (large arrows). **(B)** After i.v. delivery, GFP-labeling was associated with blood vessels (small arrows) as well as isolated neurons (large arrow) and astrocytes (arrowhead). Scale bar: 50 μm.

**Figure 10:**
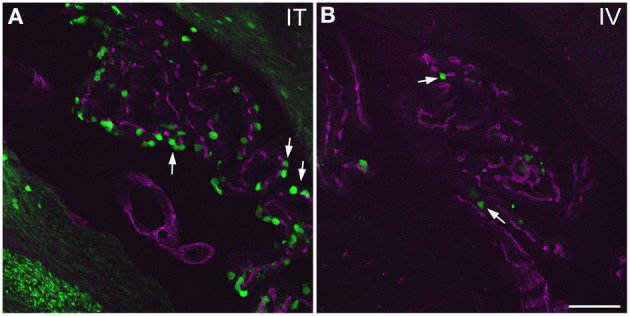
**GFP-ir in choroid plexus of the fourth ventricle**. GFP-ir (green) is present in many epithelial cells (examples indicated by arrows) after i.t. delivery **(A)** whereas labeling after i.v. delivery is limited **(B)**. Blood vessels within the choroid plexus are visualized using CD31 (purple). Scale bar: 100 μm.

Expression of GFP in the CNS was examined using immunohistochemistry across several brain regions 3 weeks after intravenous (i.v.) or intrathecal (i.t.) delivery of AAV9-GFP (Figures 6–10). Overall, i.v. delivery of vector resulted in substantially lower GFP-ir in brain compared to i.t. delivery. In i.v. treated mice, low-intensity GFP-ir was seen in isolated neurons throughout the brain without an apparent association with specific CNS regions (Figures 3G, 7A2-IV, 9B). GFP labeling was also present in astrocytes and outlined CNS blood vessels following i.v. treatment (Figure 9B).

The localization of GFP-ir in brains of i.t. treated mice is described below. All brain regions where GFP-ir was observed in neuronal cell bodies are listed in Table 1. Unlike the scattered pattern of neuronal labeling seen after i.v. delivery, in brains from i.t. treated mice the majority of GFP-positive neurons were seen within discrete nuclei and in some cases within subdivisions of certain nuclei. However, there were also numerous instances, particularly in cortex, where clusters of intensely labeled cells were sporadically associated with blood vessels rather than with a particular anatomical region. Astrocytes were always seen in association with blood vessels. It is unclear at present what factors govern the vasculature-related access of viral particles to CNS parenchyma.

**Table 1:** **Relative abundance of GFP-ir neurons in CNS regions that were transduced by AAV9: +, sporadic; ++, consistent intermediate; +++, consistent abundant; ++++, very abundant (e.g., cerebellum, Figure 6B, and olfactory bulb Figure 8B)**.

**CNS subdivision**	**Structure**	
Medulla and pons	gracile n.	+++
	cuneate n.	+++
	external cuneate n.	+++
	spinal n. of CnV	+++
	hypoglossal n.	+++
	nucleus of the solitary tract	+++
	area postrema	+++
	reticular formation	++
	inferior olivary complex	++
	vestibular nuclear complex	++
	cochlear nuclear complex	+++
	principal sensory n. of CnV	+
	motor n. of CnV	+
	facial nucleus	+++
	superior olivary complex	++
	pontine nuclei	+++
	nuclei of the lateral lemniscus	+++
Cerebellum	Purkinje cell layer	++++
	granule cell layer	++++
Midbrain	inferior colliculus (external cortex and dorsal n.)	++
	superior colliculus (internal gray layer, medial zone of deep gray layer)	++
	ventral tegmental area	+
	substantia nigra, reticulata (ventral portion only)	++
	substantia nigra, compacta (rostral vntromedial portion)	++
	interpeduncular nucleus (caudal and lateral divisions)	++
	parabrachial nuclear complex	+
Thalamus	medial geniculate n.	+++
	lateral geniculate n.	+
	lateral posterior n.	+
	ventrolateral n.	+
	ventromedial n.	+
	anterior thalamic nuclei	+
Hypothalamus	mammillary nuclear complex	++
	arcuate n.	+++
	lateral hypothalamus	+++
	tuber cinereum	++
	paraventricular n.	+
	ventromedial n.	+
	suprachiasmatic n.	++
	supraoptic n.	++
Forebrain	hippocampal formation	++++
	amygdaloid complex	+++
	striatum	+
	nuclei of the diagonal band	+++
	septal nuclei	+++
	medial preoptic area	++
	ventral pallidum	++
	islands of Calleja	+++
	cortex (sporadic clusters of neurons)	++
	olfactory bulb	++++

Neuronal GFP labeling was absent or inconclusive in regions not included in the table. The abundance of labeled neurons varied between animals at 3 weeks, but for each animal the relative abundance in different regions was consistent. Figures 6–8 illustrate examples from animals with higher level of labeling.

#### Medulla, pons, and cerebellum

Within cranial nerve (Cn) nuclei, GFP-ir was abundant in gracile, cuneate (Figures 6B,C), external cuneate, spinal (Figures 3A,B, 6B,C), and chief sensory trigeminal nuclei as well as in the nucleus of the solitary tract (Figures 3E,G). Labeling was also present in vestibular and cochlear nuclei and in some motor nuclei of cranial nerves. GFP-ir in neurons and fibers appeared to be more abundant in cochlear nuclei compared to vestibular and, within the vestibular nuclear complex, more abundant in caudal than in rostral regions. In motor nuclei, GFP-ir was most prominent in neurons of the facial nucleus, followed by hypoglossal, whereas neurons of the trigeminal motor nucleus were faintly labeled. We could not establish conclusively whether GFP-ir was present in the mesensephalic trigeminal nucleus, dorsal motor nucleus of cranial nerve X (CnX), and the nuclei of CnXI, CnVI, CnIV, and CnIII. In the medulla and pons, GFP-ir cell bodies were also found in the area postrema, inferior olivary nuclei, the superior olivary complex, nuclei of the lateral lemniscus, and pontine muclei, which are shown in Figure 7B1. Labeling in the raphe nuclear complex was not conclusively detected. GFP-ir was observed in all layers of cerebellar cortex (Cb; Figure 6B). Fiber-like labeling, presumably originating from Purkinje cells, was present in the deep cerebellar nuclei (not shown), but we saw no evidence of labeling of neurons within the nuclei.

#### Midbrain

In midbrain, GFP-ir was most prominent in clusters of neurons in layer 2 of the inferior colliculus, and neurons in the internal gray layer of the superior colliculus (Figure 7A1). In the substantia nigra GFP-ir neurons were noted in the ventral region of pars reticulata at caudal levels and at more rostral levels neurons were also present in the ventromedial portion of pars compacta. Labeling was also observed in the interpeduncular nucleus and parabrachial nucleus and to a lesser extent the ventral tegmental area. All other regions of midbrain, including the periaqueductal gray and the red nucleus, were devoid of GFP-ir neurons.

#### Thalamus, hypothalamus, and pituitary

With the exception of the medial geniculate nucleus (MGN), which contained many GFP-positive neurons in all subdivisions (Figure 7B2), relatively little GFP labeling was observed in the caudal nuclei of the thalamus. Faintly labeled neurons were noted in lateral geniculate and lateral posterior thalamic nuclei as well as in the region of ventroposterior lateral and medial nuclei. In the anterior thalamic nuclei, there were many GFP-ir fibers and a few neuronal cell bodies (Figure 7D1). Axons of the mammillo-thalamic tract (mt) could be observed crossing into the anterior nuclear complex of the thalamus (Figure 7D1). In the hypothalamus, GFP-ir neurons and nerve fibers were observed in a number of nuclei including portions of the mammillary nuclear complex, lateral hypothalamus (Figure 7C1), and arcuate, suprachiasmatic, paraventricular (PeV), and supraoptic (SON) nuclei (PeV and SON shown in Figure 7D1). Substantial GFP expression was also found in the pituitary gland (Figure 8D). Labeling in the anterior pituitary (^∧^ in Figures 8D,E) was comparable between i.t. and i.v. delivery, however, labeling in the posterior pituitary (+ in Figures 8D,E) was consistently more intense in animals that received i.t. vector. The increase in posterior pituitary expression associated with i.t. delivery corresponded well with labeling observed in the paraventricular (PeV) and supraoptic nuclei (SON) of the hypothalami of i.t. treated animals.

#### Hippocampal formation and amygdaloid complex

There was intense GFP-ir in the presubiculum (PrS; Figure 7A2-IT), parasubiculum, and subiculum. In the caudal portions of the dentate gyrus (DG), many neuronal cell bodies were observed in the polymorph (PoDG) and granular cell (GrDG) layers. The molecular layer (Mol) of the dentate gyrus and the stratum lacunosum-moleculare (LMol) of the hippocampus were clearly defined by GFP-ir both in the caudal-ventral (Figure 7B2) and rostral-dorsal (Figure 7C2) portions of the hippocampal formation. However, the rostral-dorsal hippocampus contained very few GFP-positive neurons compared to more caudal and ventral regions. GFP-ir was also noted in fibers entering the alveus of the hippocampus (alv; Figure 7B2) and well as fibers within the fornix (f) and the hippocampal commissure (hc) (Figure 7D1). In addition to hippocampus, GFP-ir was observed in portions of the amygdaloid complex (not shown).

#### Cortex, forebrain, and olfactory bulb

In the forebrain GFP-ir was prominent in the islands of Calleja, nuclei of the diagonal band, ventral pallidum, preoptic area, and septal nuclei (not shown). Labeling of neuronal cell bodies was not seen in the nucleus accumbens and was limited in the striatum. Intense GFP labeling was observed in the olfactory tubercle (not shown) and olfactory bulbs (Figure 8B). GFP-ir was found throughout the olfactory bulb in nerve fibers, neuronal cell bodies and astrocytes, but was most concentrated in the medial portions of the glomerular (Gl) and external plexiform (EPl) layers. In cortex, GFP-ir neurons and astroglia were found in clusters that were often very near to larger blood vessels (see ^*^ in Figures 7B2,D2-IT; small arrows in Figure 10A). This association was particularly striking near the surface of the cortex. The clusters appeared to be most prominent in entorhinal, retrosplenial, and cingulate cortex. With i.v. delivery, isolated GFP-positive astrocytes and neurons were also in proximity to labeled blood vessels (Figure 10B), however, association with large blood vessels near the cortical surface was not observed.

#### Choroid plexus

**Figure 11:**
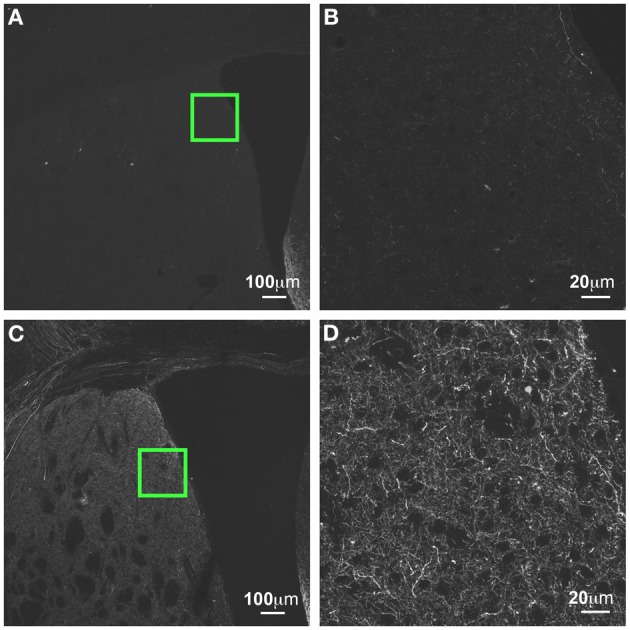
**GFP-ir in striatum at 3 weeks compared to 6 weeks after i.t. delivery. (A,B)** GFP-ir in striatum (CPu), 3 weeks after intrathecal delivery of AAV9-GFP. **(C,D)** GFP expression in striatum 3 weeks after intrathecal delivery of AAV9-GFP. Green insets in **(A,C)** are shown at higher magnification in **(B,D)** respectively.

Labeling of choroid plexus was seen throughout the ventricular system (third (3V) and lateral (LV) ventricles shown in Figure 7D1). GFP-ir was most frequently seen in cells whose morphology and localization relative to blood vessels was consistent with that of epithelial cells (Figure 11). These cells were more abundant in i.t. treated compared to i.v. treated mice. Other GFP-positive cell types were also noted, but their identification was not pursued in this study.

#### Time-dependent increase in GFP-ir

GFP-ir was more abundant at 6 weeks after i.t. vector administration compared to 3 weeks. The intensity of labeling and the density of labeled neurons within the regions described above appeared to be increased. We did not note presence of GFP-ir neurons in areas that were not labeled at 3 weeks. However, labeling within nerve fibers was increased throughout the brain. For example, prominent increase in neuropil-like labeling at 6 weeks relative to 3 weeks was seen in the striatum (CPu; Figure 11).

### AAV9-mediated GFP expression in peripheral organs and tissues

**Figure 12:**
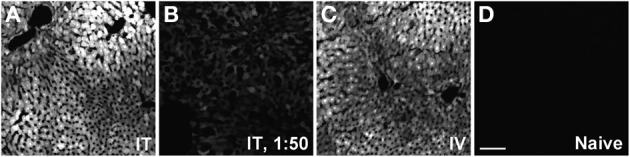
**Representative images of GFP-ir in liver 3 weeks after AAV9-GFP administration. (A)** i.t. treatment; **(B)** low-dose i.t. treatment; **(C)** i.v. treatment; **(D)** naïve. Scale bar: 100 μm.

**Figure 13:**
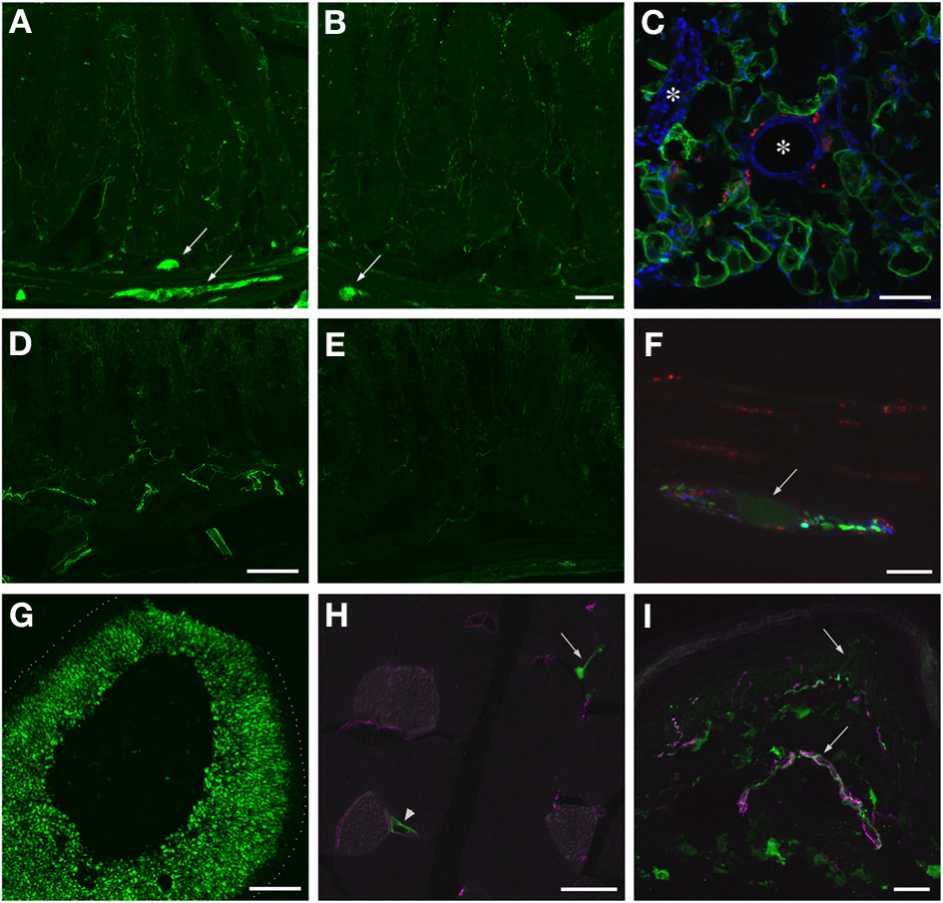
**Localization of GFP-ir in peripheral tissues after i.t. or i.v. delivery of AAV9-GFP without mannitol pretreatment. (A,B)** GFP-ir in ileum after i.t. **(A)** or i.v. **(B)** delivery. Arrows indicate enteric ganglia. Scale bar: 50 μm. **(C)** GFP-ir (green) in adipocytes associated with ileac mesentery. DAPI (blue) labeling of nuclei outlines blood vessels (^*^). CGRP-ir nerve fibers in the mesentery are shown in red. Scale bar: 100 μm. **(D,E)** GFP-ir in colon after i.t. **(D)** or i.v. **(E)** delivery. Scale bar: 50 μm. **(F)** GFP-ir (green, arrow) neuron within a myenteric ganglion in colon. GFP-, SP- (red), and CGRP-ir (blue) nerve fibers are seen within the ganglion. Scale bar: 10 μm. **(G)** GFP-ir in adrenal cortex. Dotted line indicates the edge of the tissue. Scale bar: 200 μm. **(H)** GFP-ir in nerve endings (arrow) and blood vessel (arrowhead) in thigh skeletal muscle. CD31 labeling of endothelial cells is shown in purple. Scale bar: 25 μm. **(I)** GFP- and CGRP-ir (purple) in skin. Arrows indicate a nerve bundle in dermis and an epidermal nerve fiber. Scale bar: 25 μm. **(C,F–I)** Show labeling in tissues from i.t. treated mice. The pattern of labeling in the same tissues of i.v. treated mice was similar.

GFP-ir was examined in liver, ileum, colon, adrenal gland, skeletal muscle, and skin 3 weeks after i.t. or i.v. AAV9-GFP delivery. Abundant labeling was seen in the liver of both i.t. and i.v. treated mice (Figure 12). In the ileum, GFP-ir was present predominantly within the enteric nervous system. Labeling of nerve fibers was prominent in the mucosa, submucosa, and enteric ganglia. GFP-ir was also seen within the cell bodies of some enteric neurons. The abundance and pattern of GFP labeling was comparable in ileum of i.t. and i.v. treated mice (Figures 13A,B). GFP-ir was also noted in adipocytes associated with ileal mesentery of both treatment groups (Figure 13C). In the colon, the density of GFP-positive nerve fibers was substantially higher in i.t. treated compared to i.v. treated mice (Figures 13D,E). GFP-positive myenteric neurons were observed in both groups (Figure 13F), but were fewer than in ileum. There was no apparent GFP labeling of immunocytes in lamina propria of either ileum or colon. In the adrenal gland both routes of delivery of AAV9-GFP yielded intense GFP-ir restricted to the zona fasciculata (Figure 13G). Muscle fibers within skeletal muscle were devoid of GFP-ir in both i.t. and i.v. treated mice; however, labeling was observed within nerve fibers with morphology consistent with axon endings of motor neurons (Figure 13H, arrow) as well as endings within muscle spindles (not shown). GFP-ir also appeared to be associated with some blood vessels within the muscle (Figure 13H, arrowhead). In dermis and epidermis of skin, GFP-ir was observed in nerve fibers (Figure 13I), which were more abundant in i.t treated compared to i.v. treated mice, consistent with the transduction levels of sensory neurons in the two groups. Both routes of delivery also resulted in GFP labeling of unidentified cells in the dermis (Figure 13I).

## Discussion

In the present study, we examined the biodistribution in mouse of intrathecally-delivered AAV9-GFP and compared it to the distribution achieved by an equivalent dose of viral vector administered intravenously. Intrathecal delivery yielded higher transduction efficiency in sensory neurons and the CNS. Mannitol pretreatment did not appear to enhance transduction of sensory neurons via either route of administration, as previously reported for CNS after i.v. delivery (Gray et al., 2011). Remarkably, the majority of peripheral tissues examined in this study were similarly transduced following i.v. and i.t. vector delivery, suggesting that a substantial amount of intrathecally injected viral particles are redistributed from the subarachnoid space to the systemic circulation.

### AAV9-mediated transduction of primary sensory neurons

AAV9-mediated transduction of primary sensory neurons has been previously reported in rodents and non-human primates (Hirai et al., 2012, 2014; Gray et al., 2013). However, the pattern of AAV9-driven transgene expression in sensory ganglia has not been thoroughly evaluated. Following i.v. delivery of AAV9-GFP, we observed GFP labeling only in approximately 10% of DRG neurons with or without mannitol pretreatment. In contrast, AAV9-GFP administered i.t. at the same dose demonstrated remarkable transduction efficiency in lumbar DRG. Over 90% of neurons in the L4 DRG were GFP-positive in four of the mice treated i.t. (two with and two without mannitol). The transduction level in DRG was dose-dependent as only 10% of neurons were GFP-positive following a 50-fold reduction in the dose. Based on the range of AAV9-GFP doses used in these experiments and the doses used in our previous study on AAV5 and AAV8 [approximately 3-fold lower relative to the high dose in this study; (Vulchanova et al., 2010)], we conclude that the transduction efficiency of AAV9 in sensory neurons is superior to that of AAV5 and AAV8 without a requirement for mannitol pretreatment. Moreover, although at the low dose and after i.v. delivery AAV9-GFP transduced predominantly large-diameter neurons, at the high treatment dose large-diameter as well as small-diameter peptidergic and non-peptidergic neurons were targeted. These results suggest that AAV9 viral vectors are suitable for global transduction of sensory neurons, unlike AAV5 and AAV8, which target predominantly large-diameter neurons (Vulchanova et al., 2010; Jacques et al., 2012), and AAV6, which has a preference for small-diameter cells (Towne et al., 2009). Another notable distinction between AAV9 and AAV5 and 8 is that AAV9 yielded more efficient transduction in thoracic spinal cord and ganglia.

AAV-mediated transduction of trigeminal ganglion sensory neurons has not been previously described to our knowledge. We found GFP labeling within trigeminal ganglion neurons and nerve fibers in the spinal trigeminal nucleus after both i.t. and i.v. vector delivery. As would be expected based on their rostral location relative to the site of i.t. delivery, the number of GFP-positive neurons was lower in trigeminal ganglia compared to lumbar DRG. Interestingly, although as in DRG significantly more trigeminal sensory neurons were transduced in i.t. treated compared to i.v. treated mice, the i.v. route of administration yielded significantly more transduced neurons in trigeminal ganglia compared to DRG. Further exploration of AAV9 targeting to trigeminal ganglia by systemic vector delivery in combination with the use of sensory neuron-specific promoters may identify strategies for selective gene transfer to trigeminal sensory neurons. Finally, we also noted robust transduction of sensory neurons in the nodose ganglia after i.t. vector delivery. Administration of lower doses of AAV9 vectors in the cisterna magna rather that the lumbar intrathecal space may achieve preferential gene transfer to nodose compared to spinal ganglia, providing an alternative approach for the study of the vagal sensory innervation of peripheral organs (Kollarik et al., 2010; Gautron et al., 2011). The low level of GFP-ir in the solitary tract after i.v. delivery indicates that transduction in nodose ganglia via this route was limited.

### AAV9-mediated transduction in the CNS

The distribution of GFP labeling after intrathecal AAV9-GFP delivery observed in the present study is consistent with that reported by Haurigot et al. (2013) after administration of the vector in the cisterna magna, who also found expression in cerebellar Purkinje cells, cortical neurons, hypothalamus and olfactory bulb (Haurigot et al., 2013). The rostral distribution of viral particles in our experiments was clearly dose-related, as we observed very limited GFP labeling in the brain following a 50-fold dilution of the vector. Similarly, Snyder et al. (2011) reported absence of GFP expression in the brain after intrathecal administration of a similar dose of AAV9-GFP (Snyder et al., 2011). The fact that in the present study intrathecal delivery was achieved by direct lumbar puncture in conscious subjects represents an important distinction from delivery of the vector in CSF under conditions of surgical anesthesia in rodents (Snyder et al., 2011; Haurigot et al., 2013). CSF turnover in a mouse takes place within 2 h (Rudick et al., 1982; Johanson et al., 2008), and CSF flow, and likely viral particle distribution, is impacted by heart rate, subject position, and level of motor activity. Therefore, the pattern of transgene expression may be affected by the specific conditions of intrathecal delivery.

It is notable that although GFP-ir neuropil was abundant in brains of i.t. treated mice, labeled neurons were largely restricted to selected nuclei and cortical regions. Moreover, 6 weeks after vector administration, there was a substantial increase in the density of GFP-ir nerve fibers throughout the brain compared to 3 weeks and most strikingly in midbrain, striatum, cerebellum, and hippocampus. This increase was most likely due to accumulation of anterogradely transported GFP in fine neuronal processes. Although there also appeared to be more GFP-ir neurons at the 6-week time point, neuronal cell body labeling remained localized within the same anatomical structures seen at 3 weeks. These observations suggest that neuronal transduction by AAV9 is not uniform throughout the CNS but restricted to discrete regions; however, the projection pattern of the transduced neurons appears to be widespread, resulting in global axonal localization of the transgene product. Thus, intrathecally delivered AAV9 viral vectors may be utilized for gene therapy that targets specific CNS regions by employing appropriate promoters as well as for global CNS distribution of transgenes whose function involves axonal release (e.g., enzymes, growth factors). The latter scenario is relevant for conditions such as lysosomal storage diseases, where neuropathology may be reduced through global enzyme release (Wolf et al., 2011; Haurigot et al., 2013).

The preferential transduction of specific CNS regions is likely influenced by viral tropism. Glycans containing terminal galactose have been identified as cell-surface binding moieties for AAV9 (Bell et al., 2011; Shen et al., 2011), but their availability in specific CNS regions has not been characterized. Interestingly, manipulation of cell-surface glycans altered viral AAV9 tropism (Bell et al., 2011; Shen et al., 2012), suggesting that the pattern of AAV9-mediated gene transfer is likely affected by changes in glycosylation patterns under normal and disease conditions. This possibility should be addressed in the pre-clinical development of gene therapies. In addition, our observations indicate that the vasculature may contribute substantially to CNS transduction after i.t. delivery of AAV9. Association of GFP-ir cells with blood vessels was observed throughout the CNS, but was most striking near the cortical surface of i.t. treated but not i.v. treated mice. A relationship between AAV9-mediated transgene expression and blood vessels was also noted by Samaranch et al. (2012). We speculate that access of viral particles in the CSF to the perivascular space contributes to AAV9-mediated transduction in CNS parenchyma. The pattern of AAV9-driven GFP expression in the CNS may also be influenced by anterograde and/or retrograde transport of viral particles. Samaranch et al. (2014) reported that injection of AAV9-GFP in the striatum resulted in GFP labeling of neurons in substantial nigra and cortex (Samaranch et al., 2014). In our study we noted GFP expression in neurons of specific CNS pathways. For example, GFP labeling was observed at all levels of the auditory pathway as well as in regions projecting to hippocampus (e.g., entorhinal cortex) and receiving projections from it (e.g., subiculum, mammillary nuclear complex). These observations are consistent with a contribution of axonal transport of viral particles to AAV9-mediated transgene expression. The complexity of factors influencing AAV9-mediated transgene distribution in the CNS is highlighted by the fact that regions in which GFP-positive neuronal cells bodies were limited or absent in our study, such as striatum and a large portion of the thalamus, were robustly transduced following intraparenchymal vector administration. It is possible that neurons in these areas express cell-surface receptors for AAV9 and transduction is enabled after intraparenchymal injection because of overcoming of accessibility barriers. Alternatively, the high local concentrations of vector achieved after intraparenchymal injection may enable entry of AAV9 via binding to low-affinity cell-surface binding sites.

The limited CNS distribution of GFP-ir after i.v. delivery observed in our experiments is consistent with previously reported transduction mediated by single-stranded AAV9 vector (Gray et al., 2011). However, the widespread correction of CNS abnormalities in several mouse disease models (Fu et al., 2011; Ruzo et al., 2012; Garg et al., 2013; Yamashita et al., 2013) contradicts our observations. This apparent discrepancy may be explained by several experimental variables including the type of vector (i.e., single-stranded vs. self-complementary), dose, and duration of transgene expression. It has been demonstrated that self-complementary AAV vectors result in more robust transgene expression compared to single-stranded (Gray et al., 2011) albeit at the expense of packaging capacity. Our comparison of different doses and duration of transgene expression supports the contribution of these variables in achieving widespread CNS expression. Finally, we cannot rule out the possibility that GFP expression is more widespread than the levels detectable by fluorescence. It is possible that functionally relevant transgenes are effective at lower expression levels than those required for detection of reporter gene expression.

### Evaluation of potential AAV9-GFP induced neurotoxicity

Recently, AAV9-mediated CNS expression of “non-self” proteins, and GFP in particular, has been reported to induce neuroinflammation and neurotoxicity (Ciesielska et al., 2013; Samaranch et al., 2014). Based on labeling with Iba1, we did not observe reactive microglia in association with GFP-expressing CNS regions following i.v. or i.t. vector delivery, suggesting that CNS neuroinflammation was absent under our experimental conditions. This observation is consistent with the proposal that the extent of AAV9-mediated immune activation in the CNS may be dependent on a number of factors, including levels of transgene expression (Samaranch et al., 2014). Although we did not observe signs of CNS inflammation or neurotoxicity, in DRG, and to a lower extent trigeminal ganglia, of mice given intrathecally the high treatment dose we noted mononuclear aggregates and infiltrates that appeared to be associated with neuronal damage. As GFP labeling in neurons was very robust in those DRG, it is possible that GFP overexpression resulted in immune activation in the ganglia or in disruption of protein synthesis necessary for normal cell functioning in individual neurons. A relationship between high levels of GFP expression and neurotoxicity is consistent with the observation of intensely GFP-positive cells in which Nissl staining (i.e., ribosomes) was absent. Stereological analysis appropriate for evaluation of cell loss was beyond the scope of this study. However, we noted that sample sizes and proportions of large- and small-diameter neurons were similar in ganglia from i.t. and i.v. treated mice, suggesting that the observed histopathology did not affect our quantitative analysis of proportions of GFP-positive sensory neurons and that the extent of neuronal damage may be limited. Finally, the histopathology was more limited in DRG from mice in the 6-week treatment group, which received viral vector from a different lot, raising the possibility that viral vector preparation may also be a factor. In the context of other reports of GFP-related toxicity (Ciesielska et al., 2013; Samaranch et al., 2014) and the multifactorial determinants of AAV-mediated gene transfer, the present observations call for increased vigilance in evaluating outcomes of AAV9-driven transgene expression in sensory neurons.

### Distribution of AAV9-mediated GFP expression in peripheral tissues and organs

The present study extends previous observations on the peripheral distribution of AAV9-mediated gene transfer (Zincarelli et al., 2008; Fu et al., 2011; Benkhelifa-Ziyyat et al., 2013) and is consistent with the correction of peripheral pathology after AAV9-mediated gene transfer in a mouse model of a lysosomal storage disease (Haurigot et al., 2013). Transduction in the liver was robust and equivalent for i.v. and i.t. delivery. In mice treated i.t. with a 50-fold lower vector dose, GFP-expression in liver was reduced but still appeared uniform. In contrast to these observations, Samaranch et al. (2012) reported very low GFP expression in liver of non-human primates, in which AAV9-GFP was administered in the cisterna magna (Samaranch et al., 2012). It is unclear whether this difference in peripheral transduction is due to dosing differences, type of AAV9 vector (single-stranded vs. self-complementary), or species differences in the extent to which AAV9 vectors are redistributed to the systemic circulation following delivery in the CSF. The potential for such substantial species differences in viral vector distribution between rodent and primate pre-clinical models should be given serious consideration in the translational development of gene therapies.

Our findings of extensive labeling in the enteric nervous system are in agreement with a previous description of transduction in enteric ganglia (Fu et al., 2011). Interestingly, while the pattern of GFP-ir was similar in the ileum of i.t. and i.v. treated mice, in the colon GFP-ir was substantially more abundant following i.t. vector delivery. Furthermore, transduction of intrinsic enteric neurons was less frequent in colon compared to ileum following both routes of delivery. These observations suggest that the ileum is more accessible than colon to systemically available AAV9 viral particles for reasons that are presently unclear. Additionally, the extensive GFP labeling in the colon of i.t. treated mice is likely to be associated with nerve fibers of extrinsic sensory neurons whose cell bodies are located within DRG as we have previously described for AAV8-mediated GFP expression in colon (Schuster et al., 2013). It is also likely that a proportion of the GFP-ir nerve fibers in colon as well as ileum are vagal primary afferents since in the present study we noted transduction of sensory neurons in nodose ganglia. In addition to GFP labeling within the enteric nervous system, non-neuronal GFP-positive cells were occasionally encountered in both ileum and colon. The identity of these cells remains to be determined. While evaluating GFP labeling in the ileum, we noted transduction of adipocytes with the associated mesentery. Although GFP labeling of more prominent depots of white adipose tissue was not examined in the present study, this observation raises the possibility that AAV9 vectors may be useful for gene transfer to adipocytes.

Our analysis of AAV9-mediated transduction in the periphery included the adrenal gland, which to our knowledge has not been previously evaluated. A similar pattern of GFP expression was observed after i.t. and i.v. delivery of the vector. Interestingly, GFP-ir appeared to be restricted to the zona fasciculata of the adrenal cortex with minimal labeling in the other layers of the cortex or in the adrenal medulla. A similar pattern of labeling with reduced intensity was observed at the lower treatment dose used in this study. These observations suggest potential utility of AAV9 for genetic manipulation of glucocorticoid-producing endocrine cells.

Under the present experimental conditions, transduction of skeletal muscle was not observed, in contrast with previous reports (Zincarelli et al., 2008; Foust et al., 2009; Fu et al., 2011; Elmallah et al., 2014). This discrepancy could be due to differences in type of AAV9 vector (i.e., single-stranded vs. self-complementary), dose, or time point of tissue evaluation. The observation of GFP-positive nerve endings in skeletal muscle is consistent with GFP expression in spinal motor and sensory neurons. Similarly, the presence of GFP-positive nerve fibers in dermis and epidermis is in agreement with the observed transduction of sensory neurons. The identity of other GFP-positive structures in the dermis is presently unknown. We speculate that they may correspond to blood vessels as instances of GFP labeling of blood vessels were also observed in skeletal muscle and intestine. These findings may reflect AAV9-mediated transduction of endothelial cells and require further investigation.

## Conclusions

The present analysis of the biodistribution of single-stranded AAV9-GFP following intrathecal or intravenous delivery yielded several important observations related to AAV9-mediated gene transfer in the central and peripheral nervous system. Intrathecal administration of AAV9-GFP resulted in global transduction of lumbar DRG neurons as well as efficient transduction of trigeminal and nodose ganglion neurons. The adverse effects of transgene expression noted in DRG neurons suggest that the possibility for tissue-dependent, titer-and time-related toxicity associated with gene transfer should be considered and assessed on a case-by-case basis for each serotype and gene employed. Intrathecally delivered AAV9-GFP yielded broad CNS expression of GFP that was substantially elevated relative to intravenous delivery. The pattern of CNS transduction is not uniform and is likely to be influenced by factors such as axonal transport and interactions of viral particles with elements of the blood-brain barrier. Finally, in several peripheral tissues transduction was comparable following i.t. or i.v. delivery, suggesting substantial systemic redistribution of i.t. injected AAV9-GFP. In summary, the present study demonstrates that the intrathecal route of administration results in AAV9-mediated gene transfer to a broad spectrum of discrete central and peripheral nervous system regions as well as peripheral organs in a dose-dependent and time-dependent manner.

## Author contributions

Daniel J. Schuster participated in perfusions and dissections, immunohistochemistry, imaging, cell quantification, and performed the intravenous injections. He contributed to data analysis, preparation of figures, interpretation of the results, and edited the manuscript. Jaclyn A. Dykstra participated in perfusions and dissections, performed the histopathological analysis, and contributed to data analysis and interpretation. Maureen S. Riedl participated in perfusions and dissections, histochemical analyses, interpretation of results, and edited the manuscript. Kelley F. Kitto conducted all intrathecal injections of vector and is responsible for the quality assurance of this key technique. Lalitha R. Belur initiated the studies, contributed to the experimental design, and edited the manuscript. R. Scott McIvor contributed to the experimental design, edited the manuscript, and supported the studies. Robert P. Elde contributed to experimental design and data interpretation. Carolyn A. Fairbanks contributed to the experimental design, edited the manuscript, and supported the studies. Lucy Vulchanova participated in perfusions and dissections, immunohistochemistry, imaging, and cell quantification. She contributed to experimental design, data analysis, cell quantification, preparation of figures, interpretation of the results, manuscript preparation, and support of the study.

### Conflict of interest statement

The authors declare that the research was conducted in the absence of any commercial or financial relationships that could be construed as a potential conflict of interest.
